# Evaluation of echocardiography monitoring in myotonic dystrophy type 1 patients

**DOI:** 10.3389/fcvm.2025.1574885

**Published:** 2025-07-01

**Authors:** D. S. H. Bovenkerk, C. E. W. Janssen, F. M. A. Van den Heuvel, I. B. T. Joosten, D. Den Uijl, B. G. M. Van Engelen, S. M. J. Van Kuijk, R. Nijveldt, R. Evertz, C. G. Faber, K. Vernooy, G. P. Bijvoet

**Affiliations:** ^1^ Department of Neurology, School for Mental Health and Neuroscience, Maastricht University Medical Center, Maastricht, Netherlands; ^2^ Department of Cardiology, Cardiovascular Research Institute Maastricht (CARIM), Maastricht University Medical Center, Maastricht, Netherlands; ^3^ Department of Cardiology, Radboud University Medical Center, Nijmegen, Netherlands; ^4^ Department of Neurology, Donders Institute for Brain, Cognition and Behaviour, Radboud University Medical Center, Nijmegen, Netherlands; ^5^ Department of Clinical Epidemiology and Medical Technology Assessment, Maastricht University Medical Center, Maastricht, Netherlands

**Keywords:** myotonic dystrophy type 1, echocardiography, left ventricular dysfunction, routine echocardiographic follow-up, conduction abnormalities

## Abstract

**Introduction:**

Myotonic Dystrophy type 1 (DM1) is the most prevalent genetic neuromuscular disorder. The potential cardiac involvement, including arrhythmia and left ventricular dysfunction, demands routine follow-up with ECG and echocardiography, however recommendations on the interval of echocardiographic follow-up varies substantially. The aim of this study is to evaluate the long-term prevalence of LV dysfunction during echocardiographic follow-up of patients with DM1. Secondly, we aim to assess the association between structural abnormalities on echocardiography, ECG and clinical parameters.

**Methods:**

This retrospective cohort study with DM1 patients was conducted in Maastricht University Medical Center (MUMC+) and Radboud University Medical Center. All patients above 18 years old were included through the Dutch DM1 patient registry (MYODRAFT study). Patients were evaluated between January 2010 and December 2023. A total of 273 patients were included, in whom echocardiographic data was collected and correlated with clinical data, neuromuscular status and ECG parameters.

**Results:**

At baseline 20/273 (7.3%) patients had LV dysfunction (LVEF < 50% and/or LV dilatation). When newly detected LVEF <50% alone is considered then the yield of routine echocardiography follow-up was: 9/273 at baseline (3%), 2/84 at 36 months (2%), 9/184 at 72 months (5%) and 10/117 at the interval beyond 72 months (8%). The only clear correlation between ECG and echocardiography abnormalities was a widened QRS interval 125 ± 31 ms vs. 103 ± 19 ms (*p* = 0.007). This was also demonstrated in the multivariable analysis. Of the DM1 patients developing LV dysfunction, the median interval between the diagnosis of DM1 and the first echocardiogram detecting LV dysfunction was 181 months (15.1 years) with an interquartile range of 85–301 months (7.0–25.1 years).

**Discussion:**

Based on the data of this large retrospective study, the occurrence of LV dysfunction in DM1 patients is rather low (7.4%) at baseline and increases with 6.5% at 72 months follow-up. There is a significant correlation between LV dysfunction and a widened QRS interval. Which could prompt earlier echocardiographic follow up in this patient group. Routine echocardiography is useful in DM1 however the interval of echocardiography could be shifted more towards 5 years in asymptomatic patients because of the slow progressive nature of the disease.

## Introduction

Myotonic Dystrophy type 1 (DM1) is the most prevalent genetic neuromuscular disorder with a prevalence of 1 in 7,400–10,700 in Europe (1). It is characterized by progressive proximal and distal muscle weakness, facial muscle weakness and myotonia, as well as multi-organ involvement. Frequently affected organs are the heart, lungs, eyes, and the gastrointestinal system (1–3). The mean age at death for DM1 patients is 54 years (95% CI: 52–56.7) (3), often due to pulmonary (31%) and cardiac disease (29%) (3). The most common causes of cardiac death are atrioventricular blocks and ventricular arrhythmias (4, 5). The prevalence of conduction delay does not differ between subtypes of DM1 (6). Echocardiographic abnormalities [e.g., left ventricular (LV) dysfunction, LV hypertrophy, LV dilatation, mitral valve prolapse] have been reported in up to 37% of patients (7). Systolic LV dysfunction is described in around 14% of patients with DM1 (7–10) and is generally associated with clinical heart failure and a worse prognosis (11). Early detection of arrhythmias and conduction disturbances is important to prevent life threatening complications (5).

Current consensus-based care recommendations describe the need for an echocardiogram and 12-lead electrocardiogram (ECG) at baseline, and annual follow-up with 12-lead ECG in asymptomatic patients (9). The recommendations regarding interval of echocardiographic screening varies throughout literature from yearly up to every five years (9). The socio-economic effects of frequent routine testing, and the burden on these patients with often low mobility, prompts to investigate the diagnostic yield of routine echocardiographic testing. Therefore, the aim of the present study was to assess the prevalence of LV dysfunction during follow-up of patients with DM1. Secondly, we aim to assess the association between clinical parameters such as ECG findings, Cytosine-Thymine-Guanine (CTG) repeat length and structural abnormalities on echocardiography.

## Material and methods

### Study design and population

This retrospective observational cohort study was conducted at the Maastricht University Medical Centre (MUMC+) and Radboud University Medical Centre (Radboudumc), that together form the Myotonic Dystrophy Expertise Centre in The Netherlands. The Dutch DM1 patient registry (MYODRAFT study) was used to identify DM1-affected individuals older than 18 years, and all patients that had received at least one ECG and echocardiogram in the expertise centre between January 2010 and December 2023 were included in this study. All participants provided written informed consent. The study was conducted in accordance with the Declaration of Helsinki, and the research protocol was approved by the institutional Medical Ethics Committee (METC 16-4-001, approved on 18-03-2016).

### Cardiac assessment

DM1 patients were annually screened by a cardiologist with DM1 expertise. Cardiac follow-up consisted of history taking, cardiac symptom evaluation, (such as palpitations, dyspnea, dizziness, and syncope), physical examination, and 12-lead ECG. Echocardiography was routinely performed at baseline and every 2–5 years thereafter according to our DM1 protocol. The baseline measurement was defined in this study as the first echocardiogram available after 2010. Subsequently, the echocardiograms were categorized into three follow-up intervals based on time from baseline: those performed within 36 months, those performed after 36 months but within 72 months, and those performed beyond 72 months.

### Echocardiography

Echocardiograms were evaluated for the following parameters: LV ejection fraction (LVEF) and LV end diastolic diameter (LVEDD). LV dysfunction was defined as either LVEF < 50% or LV dilatation [>58.4 mm in men and >52.2 mm in women (12)]. LVEF was measured by default with the Simpson's biplane method, in case of poor acoustic apical windows Teichholz method or visual assessment was used. LVEDD was measured on the parasternal long axis view. The method used to measure LVEF varied between patients as well as during follow up. LVEF was categorized in <40%, 40%–50% and >50%.

### Electrocardiogram

For each participant, a corresponding 12-lead ECG conducted in the same year as the echocardiogram was collected. If that was unavailable, the most recent ECG was collected with a maximum of 2 years after the echocardiogram. ECGs were assessed for the following parameters: underlying rhythm, heart rate, PR interval, QRS duration and QTc time in milliseconds (ms). ECGs were considered abnormal in case of prolonged PR interval (>200 ms), widened QRS complex (>100 ms) or atrioventricular conduction disorders that were further categorized into 1st-, 2nd- degree Wenckebach, 2nd- degree Mobitz or 3rd- degree atrioventricular block.

### Neurological assessment

DM1 patients visited the neurology outpatient clinic annually. During each visit, a neuromuscular neurologist conducted history taking and physical examination to determine disease progression and muscle status. Additionally, the muscular impairment rating scale (MIRS) score was calculated and used to assess disease progression. The MIRS score is an ordinal five-point rating scale based on manually testing 11 bilateral muscle groups. The MIRS score was categorized as either high (4, 5) or low (1–3). A high score was in this study defined as severe muscle weakness indicating proximal muscle weakness, and a low score indicating minimal or only distal muscle weakness.

### Statistical analysis

Statistical analysis was performed using IBM SPSS statistics software version 25 (SPSS Inc., Chicago, IL, USA). Categorical data was described as frequencies and percentage. For parametric continuous data, results were presented as mean ± standard deviation or, in case of non-parametric data as median with interquartile range (IQR). Differences between groups were calculated using the independent-samples *t*-test for parametric continuous variables, for non-parametric data Mann–Whitney *U* test was performed. Chi-Square test was performed for categorical variables. Univariate univariable binary logistic regression, using predefined variables (age, sex, subtype of myotonic dystrophy, severe muscle weakness, and abnormalities on ECG), was used to identify predictors for having abnormalities on echocardiography. A qualified cardiologist and neurologist with DM1 expertise selected the predefined variables. When a *P*-value < 0.20 on univariable analysis was present, the variable was included in multivariable analysis. Results of logistic regression analyses were presented as odds ratio (OR) with confidence interval. *P*-values of <0.05 were considered statistically significant.

## Results

### Patient characteristics

**Figure 1:**
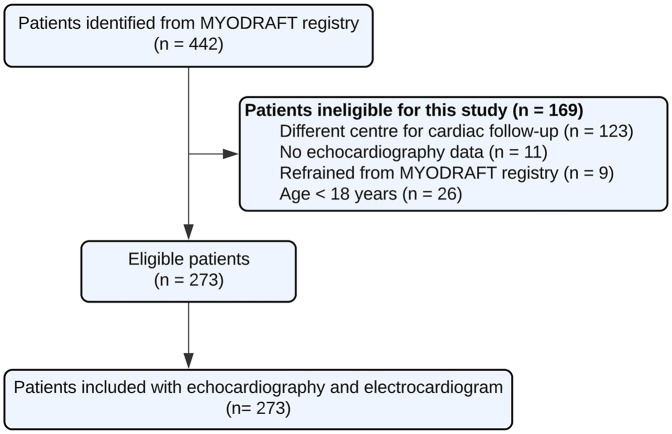
Participant flowchart.

In the MYODRAFT database, 273 DM1 adult patients were included between 2010 and 2023 who were under follow-up in the Myotonic Dystrophy Expertise Center in the Netherlands and had received echocardiographic evaluation (Figure 1). Patient characteristics at baseline are presented in Table 1. The mean age in the study population was 46 ± 14 years and the patients were followed for a median time of 66 months with an IQR of 40–95 months and a maximum of 143 months. Patients had a median of 3 echocardiograms with an IQR of 2–3 echocardiograms. The time between inclusion in the MYODRAFT database (i.e., baseline of this study) and the time of diagnosis of DM1 varied, therefore we calculated the median interval people were diagnosed with DM1 to their first echocardiography showing signs of LV dysfunction during our follow up. This interval was 181 months (15.1 years) with an IQR of 85–301 months (7.0–25.1 years). In four patients LV dysfunction was known prior to the diagnosis of DM1.

**Table 1:** Baseline characteristics.

Characteristic	Total	LV dysfunction	Normal LV function	*P*-Value
	(*n* = 273)	(*n* = 20)	(*n* = 253)	
Age (years)	46 ± 14	54 ± 15	46 ± 14	0.015
Male sex	138 (51%)	13 (65%)	125 (49%)	0.179
CTG repeat size [median (IQR)]	150 (120,200)	150 (150,200)	150 (120,200)	0.270
Severe muscle weakness^a^	58 (23%)	7 (35%)	51 (20%)	0.173
NIV indication	106 (42%)	11 (55%)	95 (38%)	0.216
Follow-up time in months [median (IQR)]	66 (40,95)	71 (52, 91)	66 (39,95)	0.723
Mean no. of echocardiograms	3 ± 1	3 ± 1	3 ± 1	0.149
ECG abnormalities	105 (38%)	14 (70%)	141 (56%)	0.112
PR interval (ms)	184 ± 34	206 ± 53	182 ± 31	0.070
QRS interval (ms)	104 ± 21	125 ± 31	103 ± 19	0.007
LVEF (%)	50 (55, 63)	45 (44, 56)	60 (56, 64)	<0.001
LVEDD (mm)	46 ± 5	52 ± 7	45 ± 5	<0.001

Continuous values are expressed as mean ± standard deviation and non parametric variables were displayed as median with a 25%–75% IQR, categorical values are expressed as frequencies (%).

^a^ Severe muscle weakness as defined by high MIRS (muscular impairment rating scale) 4–5, indicating proximal muscle weakness; NIV, non-invasive ventilation; LVEF, left ventricular ejection fraction; LVEDD, left ventricular end diastolic diameter.

### LV dysfunction at baseline

At baseline 20/273 (7.3%) patients had LV dysfunction (LVEF < 50% and/or LV dilatation) of which 16/273 (5.9%) were *de novo* findings. Baseline characteristics are shown in Table 1. The patients with LV dysfunction were significantly older than those with normal LV function at baseline (54 ± 15 vs. 46 ± 14 years old, *P* < 0.05, Table 1) and had a significantly longer QRS duration (125 ± 31 vs. 103 ± 19, *P* = 0.007). The groups with and without LV dysfunction had no significant differences regarding higher MIRS score (35.0% vs. 20.2%, *P* = 0.173) or indication for non-invasive ventilation (55.0% vs. 37.5%, *P* = 0.216), PR interval (206 ± 53 vs. 182 ± 31, *P* = 0.070) and CTG-repeat size in both groups (172.06 vs. 182.81, *P* = 0.834).

Table 2 shows univariable logistic regression that revealed significant associations of LV dysfunction with age: OR 1.04 per year; 95% CI (1.01–1.08), PR interval: OR 1.017; 95% CI (1.005–1.029), and QRS interval: OR 1.035; 95% CI (1.017–1.053). In the multivariable analysis, the only parameter that remained significant was QRS interval: OR 1.035; 95% CI (1.013–1.058).

**Table 2:** Univariate logistic regression analysis.

Variable	Univariable			Multivariable		
	OR	CI	*P*-value	OR	CI	*P*-value
Ch-Juv subtype	1.472	0.129–16,814	0.76			
Adult subtype	4.223	0.545–32.707	0.23			
CTG repeat length	1,0	0.997–1.003	0.83			
Age	1.041	1.007–1.076	0.017	1.029	0.993–1.066	0.115
PR interval	1.017	1.005–1.029	0.004	1.004	0.987–1.021	0.64
QRS interval	1.035	1.017–1.053	<0.001	1.035	1.013–1.058	0.002
Severe muscle weakness^a^	1.943	0.737–5.124	0.18	1.557	0.559–4.335	0.40

^a^ Severe muscle weakness as defined by high MIRS (muscular impairment rating scale) 4–5, indicating proximal muscle weakness; Ch-Juv, childhood-juvenile; OR, odds ratio; CI, confidence interval.

### LV dysfunction during follow-up

The interval between echocardiograms varied between patients. In 84 patients a follow-up echocardiogram was performed within 36 months after baseline, which yielded five new cases of LV dysfunction (5/84, 6.0%). In 184 patients a follow-up echocardiogram (either second echocardiogram or following) was performed between 36 and 72 months follow-up, which yielded 12 new cases of LV dysfunction (12/184, 6.5%). In 118 patients an echocardiogram (either second or following) was performed beyond 72 months after baseline (with a maximum of 143 months after baseline), which yielded another 12 new cases of LV dysfunction (12/118, 10.2%).

**Figure 2:**
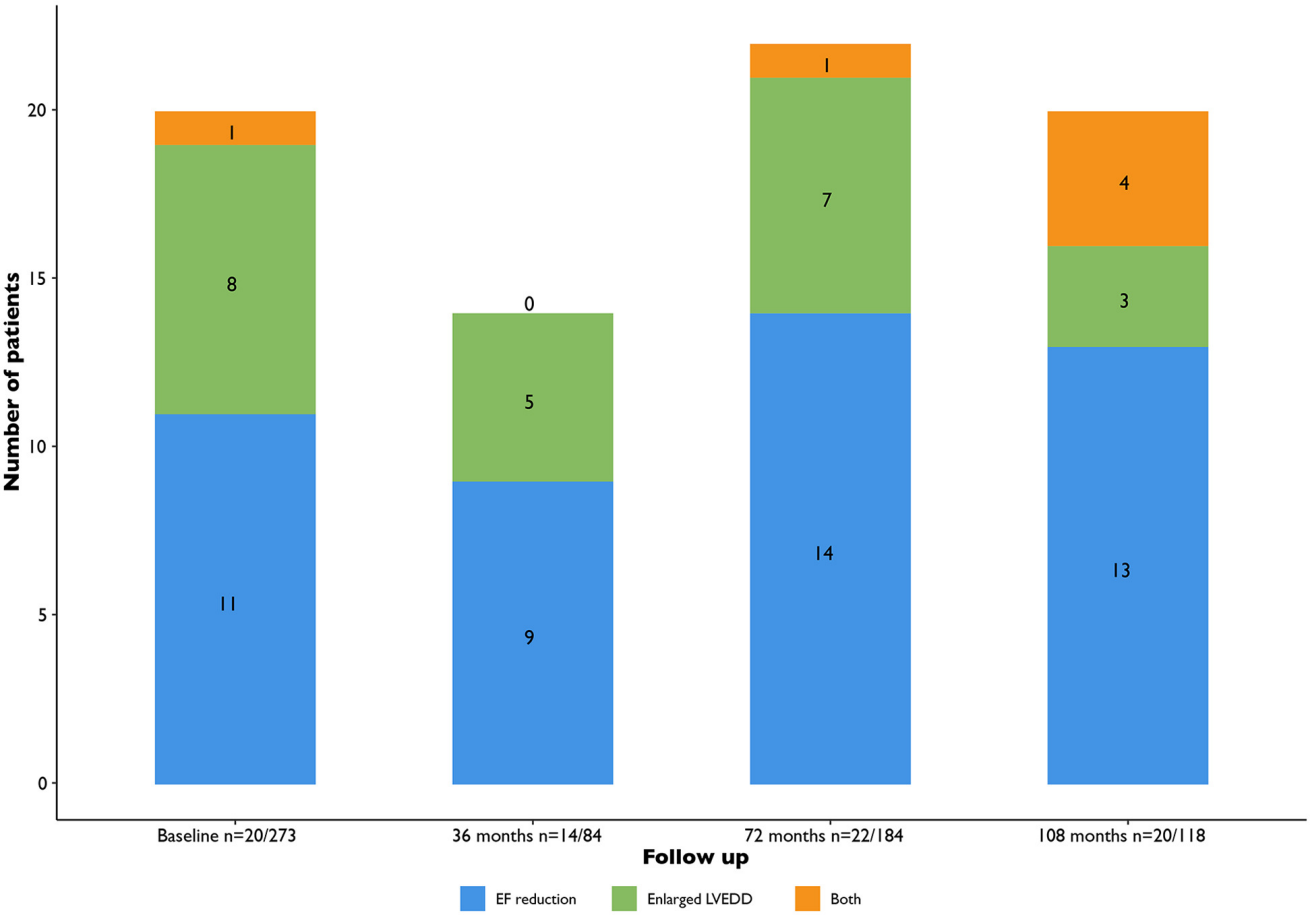
Total amount of patients with LV dysfunction (LVEF < 50% or LV dilatation). EF, ejection fraction; LVEDD, ventricular end diastolic diameter.

The total amount of patients with LV dysfunction (*de novo* findings and pre-existent combined) depending on the timing of the performed echocardiogram (within 36, 36–72, beyond 72 months after baseline) were 14/84 (16.7%), 22/184 (12.0%) and 20/118 (16.9%), respectively (Figure 2).

**Figure 3:**
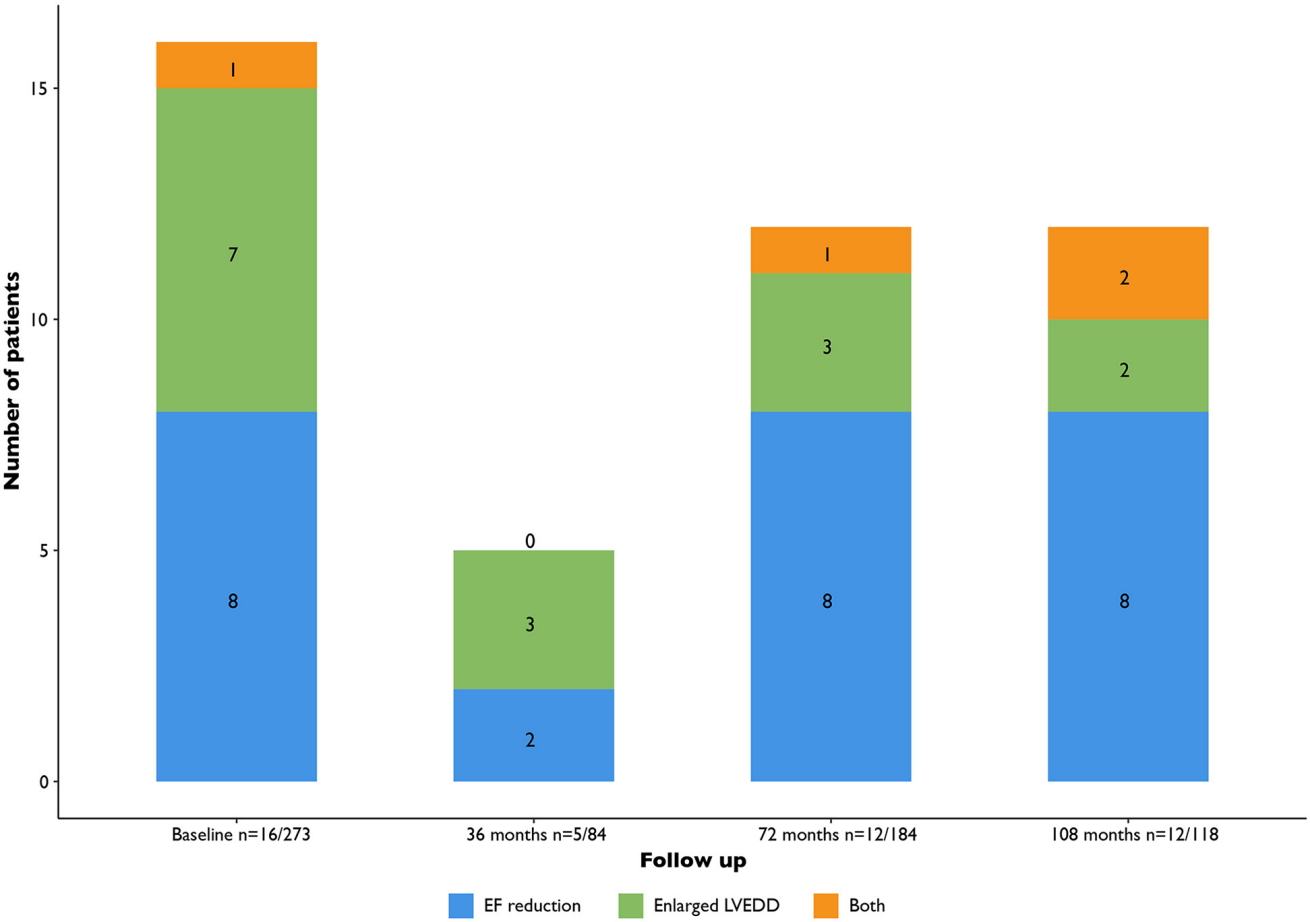
Patients with *de novo* findings of LV dysfunction (LVEF < 50% or LV dilatation). LV, left ventricular; EF, ejection fraction; LVEDD, ventricular end diastolic diameter.

When considering the yield of routine echocardiography to detect LVEF < 50% (i.e., excluding LV dilatation only from the diagnosis of LV dysfunction): nine cases at baseline (3% of patients), two new cases at 36 months (2% of screened patients), nine new cases at 72 months (5% of screened patients) and ten new cases at the interval beyond 72 months (8% of screened patients) (Figure 3).

### Clinical consequences

**Figure 4:**
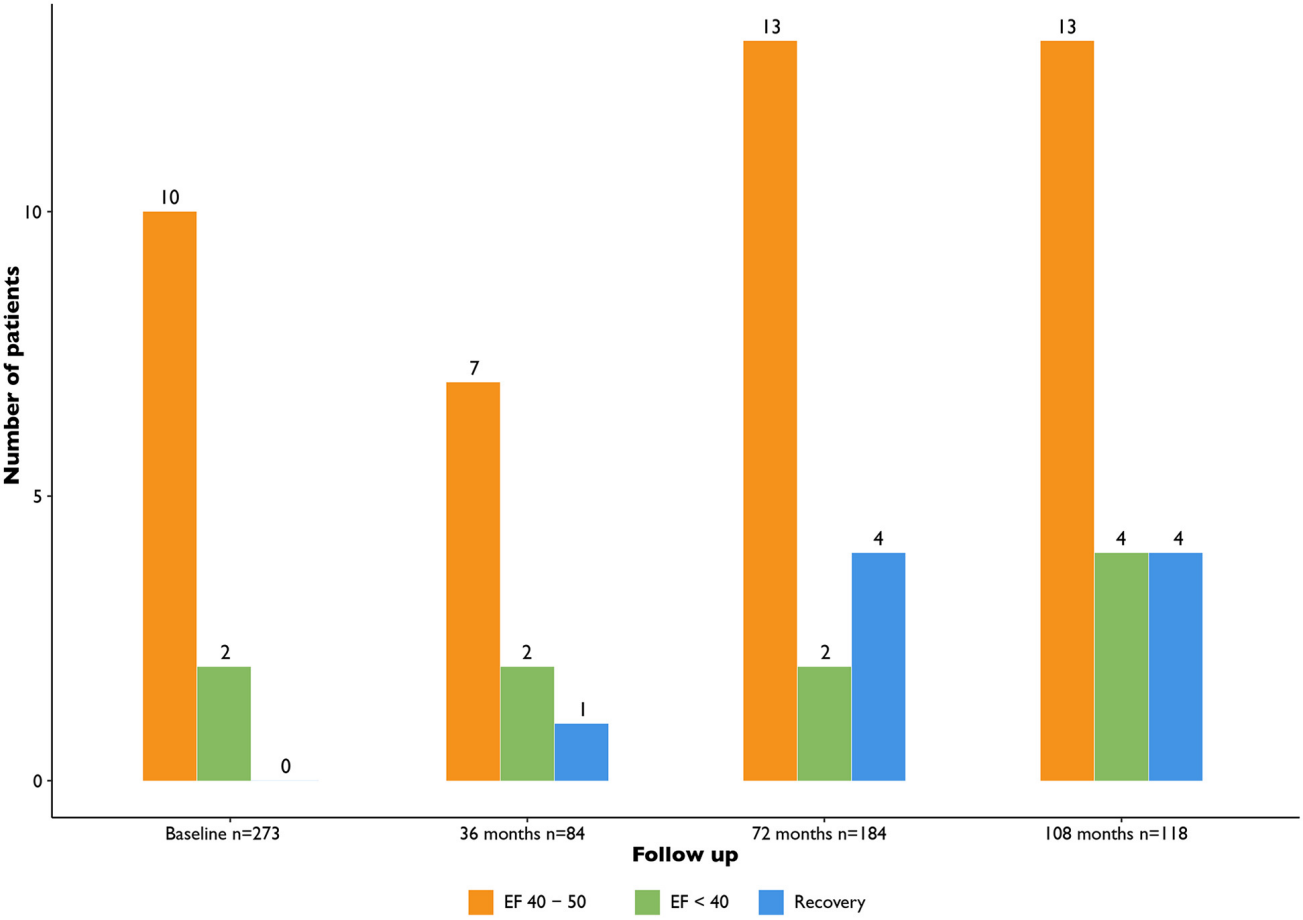
Characteristics of ejection fraction over time. EF, ejection fraction.

During follow up in this patient cohort we observed recovery of LV function in some patients (9/273, 3.3%) (Figure 4). Patients with LV dysfunction were treated following ESC guidelines for heart failure and optimal medical therapy was reached if possible. Furthermore, these patients were treated by the cardiologist with heart failure medication according to ESC guidelines. Additionally, in some cases the cardiologist was consulted in between routine screening moments. This was the case for 65/273 (27.9%) patients. In 39 (14.3%) cases this was due to ECG changes or additional Holter abnormalities in 13 (4.8%), in 9 (3.3%) cases due to clinical symptoms, in 2 (0.7%) cases due to a cardiac event (e.g., cardiac arrest). In only 2 (0.7%) cases an additional echocardiogram was deemed necessary based on the clinical problem.

### Loss to follow-up

We observed a loss to follow-up of 76 patients during our observation period, of whom 6.6% had LV dysfunction at the time of their latest echocardiogram. The reason for loss to follow-up were as follows: In seven patients, no subsequent echocardiogram was performed because of a good clinical condition. Follow-up was performed in another hospital in the Netherlands in 37 patients. 25 patients died during our observation period (9.2% of the total 276; *n* = 16 due to a non-cardiac cause, *n* = 9 due to an unknown cause). One patient declined cardiac follow-up and six patients did not have a follow-up echocardiogram due to logistic issues.

## Discussion

In this retrospective study, we evaluated the diagnostic yield of LV dysfunction shown on echocardiography, the correlation between baseline echocardiography and baseline ECG and the role for routine echocardiography follow-up in patients with DM1. The occurrence of LV dysfunction in our population is rather low with a slow progressive trend. For our cohort of 273 patients, 20 showed LV dysfunction at baseline (7.3%) and routine follow-up over a period exceeding 72 months resulted in 29 new cases of LV dysfunction. Of these 29 cases with LV dysfunction, only 16 were based on LVEF reduction alone. When we compare the percentage of *de novo* LVEF < 50% found in our cohort this is lower than found in earlier literature. Russo et al. published a systematic review including eight studies, of which two with large sample size, showing a mean prevalence of LV dysfunction 13.8%. In these studies LV dysfunction is either defined as LVEF < 50% or <55%. LVEF < 50% was present in 18.5%–20% of patients. Our larger cohort showed smaller numbers of LVEF reduction. A possible factor for these smaller numbers might be the slightly higher mean age of the population, which was 46 years in our study. In the systematic review performed by Russo et al. the age of the eight included studies was between 39 and 44 years (7). Notably, this study consists of the largest sample size to date with echocardiographic follow-up. The largest previously published study included 406 patients, however, echocardiography was only performed in a subset of 180 patients (11).

LV dysfunction has potentially serious, and clinically relevant consequences and therefore should be investigated in DM1 patients. In DM1 patients there is a risk of underreporting cardiac symptoms due to physical inactivity and cognitive dysfunction, which underlines the role of routine cardiac screening (13). The present study suggests that the slow progression towards LV dysfunction in DM1 patients and relatively low prevalence, allows the interval of routine echocardiographic follow-up in asymptomatic patient to be prolonged. Prospective studies are needed to investigate which time interval is optimal for clinical follow-up.

This study assessed the association between ECG, clinical parameters and LV dysfunction found on echocardiography. CTG-repeat size did not show a correlation with LV dysfunction, in contrast to reports in previous literature. However, ECG abnormalities were found in 70% of patients at baseline, of which conduction disorders were most prevalent (14). A substantial number of patients with LV dysfunction had either a prolonged PR interval or a prolonged QRS interval. In the multivariable analysis QRS interval remained significant signifying that this could be a predictor of LV dysfunction. We did observe that during follow-up patients with *de novo* LV dysfunction had significantly more widened QRS intervals. This remained significant in multivariable analysis and could prompt clinical decision making. However, ECG abnormalities are prevalent in most patients with DM1 and are not limited to echocardiographic abnormalities.

Previous studies have suggested that cardiac magnetic resonance imaging might be able to detect early cardiac involvement of DM1 without abnormalities on echocardiography, Holter, and ECG monitoring (15, 16). Moreover, a study performed by Guedes et al. in a small cohort (*n* = 25) demonstrated measuring global longitudinal strain with three-dimensional speckle tracking of the left atrium and LV is decreased in patients with DM1 which could be a marker of early subclinical dysfunction in these patients (17). However, based on our cohort we have not used this routinely and thus do not have the data to support any claim regarding cardiac magnetic resonance imaging in DM1.

### Limitations of this study

When interpreting the database of this real-world sample retrospectively, a standardized echocardiographic follow-up was lacking, and there was a substantial loss to follow-up. The retrospective design of this study increases the risk of bias and confounders. There was no selection bias since all patients under control in the expertise center are invited to join the MYODRAFT registry. Missing data was evaluated and if possible, extracted from the patient file. In this study we combined LVEDD and/or LVEF in the definition of LV dysfunction. The parameters are separate entities and reflect different aspects of cardiac pathology. This is a potential limitation, but we believe that taking both into consideration best reveals structural abnormalities over time in DM1. Additionally, other echocardiographic findings such as valvular disease, diastolic dysfunction and pulmonary systolic pressure were not mentioned.

## Conclusion

Based on the data of this large retrospective study, the occurrence of LV dysfunction in DM1 patients is rather low (7.4%) at baseline and increases with 6.5% at 72 months follow-up. The mean time between DM1 diagnosis and development of LV dysfunction is 20 years suggesting a slowly progressive course. Therefore, 2–5 year follow-up echocardiography is probably abundant, and the interval of routine echocardiographic follow-up might shift towards five years in asymptomatic patients. There is no significant correlation with neurological status, or CTG-repeat size to guide the timing of echocardiographic follow-up. However, the established correlation between a widened QRS interval and LV dysfunction could prompt the physician to shorten the interval between echocardiographic follow up.

## Data Availability

The raw data supporting the conclusions of this article will be made available by the authors, without undue reservation.
